# Evaluation of biomarkers for diagnosnostic decision making in patients with gout using novel mathematical model. Complex PPPM approach

**DOI:** 10.1186/1878-5085-5-S1-A58

**Published:** 2014-02-11

**Authors:** Rostyslav V Bubnov, Ivan M Melnyk

**Affiliations:** 1 Clinical Hospital Pheophania, Center for ultrasound diagnosis and interventional sonography, Kyiv, Ukraine; 2 International Research and Training Center for Information Technology and Systems NAS of Ukraine, Kyiv, Ukraine

## Introduction

Gout is a common metabolic disorder, involving liver, kidney and joints, it is met more often in men. Recently we described specific ultrasound (US) signs of gout nephropathy [1]. We suggest novel mathematical model according to which the medical process is percepted as a complex system like "black box" [2,3]. This process (disease progression) is described by some of the primary indicators (US and laboratory biomarkers). So primary indicators and output rate are stochastic in nature and presented as statistical information. The "best" mathematical model of the medical process is studied using a special algorithm for processing statistical data [2].

## The aim

was to assess the complex multiparameter evaluation of biomarkers for diagnostic decision making in patients with gout using novel mathematical model.

## Materials and methods

We considered 42 patients (35 men, 7 women) to the first group, mean age was 58 ± 4.5, whom gout was diagnosed, according to disease history, or increased levels of uric acid in blood. The average level of uric acid in patients in group was 465 µmol/L. The control group included 34 patients (16 men, 18 women), mean age 54 ± 6 years without clinical, laboratory signs of nephropathy and liver pathology. All patients underwent ultrasound examination, gray scale, Doppler and sonoelastography parameters of liver and kidneys were recorded and evaluated statistically and assessed with own model [3]. Integrated index (Y) rated ranged 0/1 for disease description and staging was calculated.

## Results

In all patients of the first group were US signs of fatty liver, in 38 patients (90%) ultrasonography signs of nephropathy were registered, significantly more frequent (P < 0.01) than in second group. The diffuse fatty liver (P < 0.01) and portal hypertension (P < 0.1) were prevailed in the first group. Transaminase increase was registered only in 7 patients (17%) of the first group. Nephropathy signs included thinning, increasing echogenicity of kidney parenchyma (P < 0.05), detection of fibrotic changes and small hyperechoic inclusions, hilly margins, anaechoic strips under the capsule, RI increasing in segmental arteries over 0.7. The revealing of nephropathy (r > 0.85) was correlated with creatinine level increase and with liver fibrosis (r > 0.8). Additional attributes were joint lesions (P < 0.01). Integrated index Y was 0.73 in first group and 0.28 in second (P < 0.01).

## Conclusions

Ultrasound can be an effective method for early detection of liver and kidneys involvement in gout patients for performing their personalized treatment. The sensitivity, specificity, positive and negative predictive value and accuracy the gout involvement of liver and kidneys using complex ultrasonography diagnostic criteria were 92.6%, 84.4%, 80%, 95%, and 91.9% respectively. Nephropathy appearance correlates with diffuse liver involvement. Integrated index Y could be reliable for disease staging and control treatment follow up.

## Recommendations

It is recommended to create the system for complex evaluation of biomarkers using suggested mathematical model, based of patient medical records for *prediction*, *personalized treatment and prevention* gout that may be applicable in still existing closed medical system, obtaining relevant extensive data.
